# Supplementary material for: Ancient genomes from Ladakh reveal 2800-year-old admixture between Tibetans and South Asians
Source: Sci Adv. 2026 Jul 24;12(30):eaeb3636. doi: 10.1126/sciadv.aeb3636 (PMC13398531; doi:10.1126/sciadv.aeb3636)
Supplement: Supplementary file 1 — Legends for tables S1 to S9 [file sciadv.aeb3636_sm.pdf]

Science Advances
AAAS

Supplementary Materials for

Ancient genomes from Ladakh reveal 2800-year-old admixture between Tibetans and South Asians

Nick Patterson *et al.*

Corresponding author: Nick Patterson, nickp@broadinstitute.org; Veena Mushrif-Tripathy, vmushrif@gmail.com; Quentin Devers, quentin.devers@cnrs.fr; David Reich, reich@genetics.med.harvard.edu

Sci. Adv. **12**, eaeb3636 (2026)
DOI: 10.1126/sciadv.aeb3636

The PDF file includes:

Legends for tables S1 to S9

Other Supplementary Material for this manuscript includes the following:

Tables S1 to S9

Supplementary Table Captions

Table S1: Details of DNA extraction, library preparation, and sequencing for 64 newly generated libraries

Table S2: Details of 10 newly generated radiocarbon dates and associated isotopic measurements

Table S3: Details of 1696 individuals used in Principal Component Analysis (Figure 2)

Table S4: Details of 1094 individuals used in the 2-source *qpAdm* analysis

Table S5: Full results for 2-source *qpAdm* models

Table S6: Details of 2685 individuals used in the 3-source *qpAdm* analysis

Table S7: Full results for 3-source *qpAdm* models

Table S8: Addition of 492 populations in turn to the right outgroups for the Brahmins_Uttrakhand.HO-Latuotanggu-China_Xinjiang_Xiaohe_BA.AG qpAdm model to probe model robustness

Table S9: Genotype matrix used to count Denisovan haplotypes
